# Changes in Quality of Life Following SARS-CoV-2 Infection Among Jewish and Arab Populations in Israel: A Cross-Sectional Study

**DOI:** 10.3389/ijph.2023.1605970

**Published:** 2023-06-12

**Authors:** Jelte Elsinga, Paul Kuodi, Haneen Shibli, Yanay Gorelik, Hiba Zayyad, Ofir Wertheim, Kamal Abu Jabal, Amiel Dror, Saleh Nazzal, Daniel Glikman, Michael Edelstein

**Affiliations:** ^1^ Azrieli Faculty of Medicine, Bar-Ilan University, Safed, Israel; ^2^ Department of Medical Microbiology and Infection Prevention, Amsterdam UMC, Amsterdam, Netherlands; ^3^ The Baruch Padeh Medical Center, Poriya, Poriah, Israel; ^4^ Ziv Medical Center, Safed, Israel; ^5^ Galilee Medical Center, Nahariya, Israel

**Keywords:** COVID-19, quality of life, inequalities, Israel, observational study

## Abstract

**Objectives:** The long-term impact of COVID-19 on health inequalities is under-researched. We investigated changes in health-related inequalities following SARS-CoV-2 infection between the Jewish majority and the Arab/Druze minority in Israel.

**Methods:** Patients with a positive SARS-CoV-2 RT-PCR test processed from one of the Northern-Israeli government hospitals between 03/2021 and 05/2022 were invited to participate. We collected socio-demographic, COVID-19-related, and health-related quality of life (HRQoL) information using a validated questionnaire. We compared pre- and post COVID-19 HRQoL changes between Jews and Arabs/Druze, up to 12+ months post-infection using an adjusted linear regression model.

**Results:** Among the 881 included participants the average post-COVID HRQoL score was lower among Arabs/Druze than Jews (0.83 vs. 0.88; *p* = 0.005). Until 12 months post-infection, HRQoL changes were similar for Arabs/Druze and Jews. After 12 months, HRQoL dropped significantly more among Arabs/Druze than among Jews (0.11 points difference between the groups; *p* = 0.014), despite adjusting for socioeconomic variables.

**Conclusion:** 12 months post-infection, COVID-19 affected the HRQoL of Arabs/Druze more than Jews, with the gap not fully explained by socio-economic differences. The COVID-19 pandemic may widen pre-existing long-term health inequalities.

## Introduction

With 754 million cases and 6.8 million reported deaths as of February 2023, the COVID-19 pandemic has caused a burden of disease unprecedented for an infectious agent in modern times [1]. Beyond the acute effects, the long-term health impact of the pandemic remains poorly understood. Evidence suggests that 13% of infected individuals still report symptoms attributable to post-SARS-CoV-2 infection, 90–150 days after their acute infection [2]. Patients report a wide range of symptoms from multiple body systems, with fatigue, breathlessness, arthralgia, sleep difficulties and chest pain as predominant symptoms [3]. Post-acute COVID-19 symptoms have been associated with decreased quality of life (QoL), mainly investigated in clinical settings [4, 5]. Although the terms “long-COVID” or post-acute sequelae of COVID-19 (PASC) are frequently used, the phenomenon lacks a specific case definition.

In October 2021, the World Health Organisation (WHO) issued an evidence brief describing how health inequalities that were impacted negatively in the SARS-CoV-2 pandemic [6]. Infection incidence, hospitalization and mortality due to SARS-CoV-2 were higher for, amongst others, socio-economically disadvantaged populations, ethnic minorities and conflict-affected populations [6], contributing to an increase in health disparities documented in multiple countries [7, 8].

As of 2022, Israel had an estimated population of 9.45 million, of which 73.9% were Jewish and 21.1% were Arab (including the Druze minority) and 5% belonged to other smaller minorities such as non-Arab Christians, Circassians and those without a registered religion [9]. Most municipalities in Israel are either predominantly Jewish or Arab, with a minority of mixed municipalities. Among the 20% of households with the highest income, 96% were Jewish or other minorities and 4% were Arab, while the 20% of lowest-income households had an Arab overrepresentation (27%) [10]. Socioeconomic disparities in Israel translate to health inequalities: life expectancy is 3–4 years lower in the Arab population compared with the Jewish population [11]. With regards to COVID-19, the Arab population reported higher incidence, excess mortality and lower vaccine coverage compared with the Jewish population. A substantial part of 2020 and 2021 lockdowns were enforced at the municipality level according to a weekly calculation of infection parameters [12]. Because Arab municipalities experienced a higher disease burden, they also experienced longer and more frequent COVID-19-related restrictions.

Most of the available evidence referring to health inequalities associated with the COVID-19 pandemic, in Israel and elsewhere, investigates the acute effects of the infection or programmatic disparities and are based on infection, admission rate [6], or vaccination. Evidence suggests that in Israel there is a greater likelihood of experiencing post-acute COVID symptoms among low-income and among marginalized groups [13]. There is less evidence available on the differential long-term impact of COVID-19 on Health-Related Quality of Life (HRQoL) and its effect on inequalities. To our knowledge, changes in quality of life during the COVID-19 pandemic have not been previously studied among different groups comprising the Israeli population. We aimed to describe changes in HRQoL following SARS-CoV-2 infection among Jewish and Arab populations in Israel to determine whether the COVID-19 pandemic has impacted health inequalities between these two population groups.

## Methods

### Study Design and Participants

We conducted a cross-sectional study using an online questionnaire that collected information about participants’ physical, mental and social health and wellbeing. All individuals over 18 years old who performed a reverse transcription polymerase chain reaction (RT-PCR) for SARS-CoV-2 in one of the government hospitals in the Northern district of Israel (Ziv Medical Centre, Padeh-Poriya Medical Centre and Galilee Medical Centre) were eligible to enter the cohort regardless of the test result. Between 03 January 2021 and 08 May 2022, all individuals for whom a valid phone number was available were invited through Short Message Service (SMS), which included a questionnaire link. Although SMS were sent in batches to all participants, the date of infection varied for each participant from the beginning of the pandemic to shortly before receiving the questionnaire, and as such the dataset includes a range of follow-up times between infection and answering the questionnaire. Questionnaires were available in Israel’s frequently spoken languages, including Hebrew, Arabic, English and Russian. If invited individuals did not respond, two reminders were sent via SMS. Individuals over the age of 18 who reported a PCR test positive for SARS-CoV-2, and answered questions on ethnicity, date of infection and HRQoL were included.

### Population Groups in Israel

Categorising Israeli population groups is complex and mainly performed based on a combination of ethnicity and religious practices. The Jewish population can further be subdivided into “secular,” “religious,” “traditional” and “ultra-orthodox.” The largest ethnic minority is Arab which can be subdivided into non-Bedouin Muslim, Christian and Bedouin. Other minorities include, amongst others, Druze and Circassian [14]. In this study, similarly to Ministry of Health publications on health inequalities, the Jewish population includes all Jewish populations regardless of the level of religious observance and the Arab population includes the Bedouin, non-Bedouin Muslim, Christian and Druze minorities. The “Other” category includes foreign residents and non-Arab Christians mainly of Russian origin. Because the populations mainly live within Jewish-majority population centres, we have included them into the Jewish category.

### Measurement Tools

The questionnaire comprised an Israel-adapted version of the International Severe Acute Respiratory and emerging Infection Consortium (ISARIC) COVID-19 study instrument that was developed by an international, interdisciplinary working group [15]. Furthermore, HRQoL was assessed using the EuroQol-5 Dimension-5 Level (EQ-5D-5L) questionnaire, a validated tool that assesses HRQoL in five dimensions: mobility, self-care, usual activities, pain/discomfort and anxiety/depression [16]. Each dimension is measured with a five-point Likert scale. A country-specific weighted HRQoL score (index score) can be calculated using validated value sets, that generally range from 1.00 (highest HRQoL) to less than 0. Israel does not have a value set validated and none of the value sets were validated in a country comparable to Israel. After consultation with the EuroQol group, the most used value set (US value set) was used for this study.

### Data Collected

Socio-demographic variables that were obtained in the questionnaire included sex, age, ethnicity, place of residence, education, monthly income household and marital status. SARS-CoV-2 specific questions included vaccination, previous SARS-CoV-2 infections and outcome of the RT-PCR test, symptoms and EQ-5D-5L at the time of the interview, and retrospectively assessed EQ-5D-5L before SARS-CoV-2 infection. The time of the interview was collected to adjust for seasonal variation in HRQoL [17].

### Outcomes

We calculated the HRQoL score for each participant before and after the SARS-CoV-2 infection. In addition, we calculated the change in score (delta HRQoL). A positive delta HRQoL score indicated an improvement in HRQoL after SARS-CoV-2 infection; a negative delta HRQoL score indicated a worsening HRQoL after SARS-CoV-2 infection. We categorised participants according to the time elapsed between infection and answering the questionnaire. The different strata included: 0–30 days, 31–90 days, 3–6 months, 7–12 months and more than 12 months.

### Statistical Analyses

Data cleaning and statistical analyses were performed using R version 4.1.0. Comparisons of means were performed with a t-test for normally distributed continuous variables. Proportions between groups were compared using a Pearson’s Chi-squared Test (with Yates’ continuity correction) or a Fisher’s Exact Test if suitable. The association between HRQoL scores and delta HRQoL scores and population groups was tested using linear regression models, adjusting for relevant sociodemographic factors. *p*-values lower than 0.05 were considered statistically significant. It is worth noting that because each time point represents different individuals according to the time elapsed between infection and answering the questionnaire, we only compared populations within time points and each time point to its own baseline. The study design did not allow to calculate changes in HRQoL between time points.

### Ethical Considerations

Ethical approvals for this study were obtained from the Ziv Medical Centre, Padeh-Poriya Medical Centre, and Galilee Medical Centre ethical committees, reference numbers; 0007-21-ZIV, 009-21-POR, and 0018-21-NHR, respectively.

### Funding Source

This study was partly funded from a donation from the Harvey Goodstein Charitable Foundation. Funders had no input into study design and the collection, analysis or interpretation of data.

## Results

**FIGURE 1:**
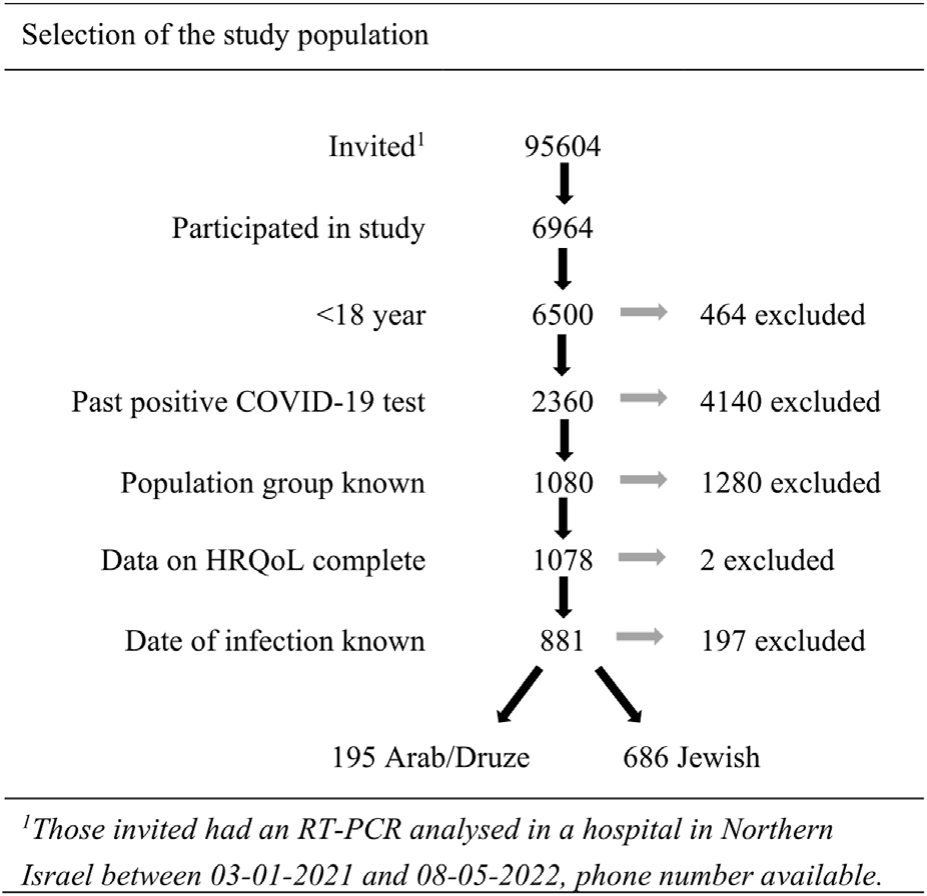
Participant selection flowchart (Israel, 2020–22).

Between 03 January 2021 and 08 May 2022, 95,604 persons were invited and 6964 (7.3%) consented to participate in the prospective cohort study that aimed at understanding SARS-CoV-2 sequelae and its impact on quality of life. Within this cohort study, the study population was selected based on the previously mentioned inclusion criteria, resulting in 881 eligible participants (Arab/Druze: 195; 22.1%—and Jewish: 686 (of which 17 belonging to the “other” category; 77.9%) (Figure 1).

Table 1 describes the sociodemographic characteristics of the two population groups. The Jewish population was older, lived more in rural areas, and had higher household income, reflecting differences within the broader population. Jewish participants also had a slightly shorter time to follow-up after their SARS-CoV-2 infection than the Arab/Druze population (mean: 203 days, 95% CI: 189–216 days vs. mean: 257 days, 95% CI: 233–282; *p* < 0.001). There was no difference in the two groups in terms of gender, proportion vaccinated and proportion reporting symptoms post infection.

**TABLE 1:** Sociodemographic and SARS-CoV-2 infection characteristics of the study population (Israel, 2020–22).

	Jewish (*N* = 686)*	Arab/Druze (*N* = 195)	Total (*N* = 881)	*p*-value
Sex				0.300^a^
Female	470 (68.5%)	122 (62.6%)	592 (67.2%)	
Male	215 (31.3%)	73 (37.4%)	288 (32.7%)	
Other	1 (0.1%)	0 (0%)	1 (0.1%)	
Age (years)				<0.001
18–40	243 (35.4%)	102 (52.3%)	345 (39.2%)	
41–60	298 (43.4%)	75 (38.5%)	373 (42.3%)	
Over 60	145 (21.1%)	18 (9.23%)	163 (18.5%)	
Place of residence				<0.001^a^
Urban	373 (55.8%)	104 (56.2%)	477 (55.9%)	
Rural	292 (43.7%)	70 (37.8%)	362 (42.4%)	
Other	3 (0.4%)	11 (6.0%)	14 (1.6%)	
Education				0.347
Other, incl. elementary school	32 (4.95%)	11 (6.15%)	43 (5.21%)	
High school	257 (39.8%)	60 (33.5%)	317 (38.4%)	
Bachelor	194 (30.0%)	64 (35.8%)	258 (31.3%)	
MSc/PhD	163 (25.2%)	44 (24.6%)	207 (25.1%)	
Monthly income household				0.001
<8,000 NIS	175 (30.9%)	64 (40.5%)	239 (33.0%)	
8,000–15,000 NIS	207 (36.6%)	66 (41.8%)	273 (37.7%)	
>15,000 NIS	184 (32.5%)	28 (17.7%)	212 (29.3%)	
Employment				0.605^a^
Part-time job	426 (62.4%)	118 (60.8%)	544 (62.0%)	
Full-time job	30 (4.4%)	5 (2.6%)	35 (4.0%)	
Military, national or social service	13 (1.9%)	3 (1.6%)	16 (1.8%)	
Caregiver/unemployed/incapacitated/retired/other	214 (31.3%)	68 (35.1%)	282 (32.2%)	
Marital status				0.072^a^
Married	534 (78.9%)	153 (78.9%)	687 (78.9%)	
Single	128 (18.9%)	41 (21.1%)	169 (19.4%)	
Other	15 (2.2%)	0 (0%)	15 (1.7%)	
SARS-CoV-2 vaccination status				0.619^b^
Not vaccinated	145 (21.2%)	45 (23.2%)	190 (21.6%)	
Vaccinated	539 (78.8%)	149 (76.8%)	688 (78.4%)	
Time of interview^c^				<0.001
2021 July–August	99 (14.4%)	34 (17.4%)	133 (15.1%)	
2021 November–December	201 (29.3%)	84 (43.1%)	285 (32.3%)	
2022 March–May	386 (56.3%)	77 (39.5%)	463 (52.6%)	
Time to follow-up after SARS-Cov-2 infection				<0.001
0–30 days	77 (11.2%)	9 (4.6%)	86 (9.8%)	
31–90 days	192 (28.0%)	39 (20.0%)	231 (26.2%)	
3–6 months	140 (20.4%)	33 (16.9%)	173 (19.6%)	
7–12 months	142 (20.7%)	58 (29.7%)	200 (22.7%)	
12 months<	135 (19.7%)	56 (28.7%)	191 (21.7%)	
Symptoms post SARS-Cov-2 infection				0.321
No	225 (32.8%)	56 (28.7%)	281 (31.9%)	
Yes	461 (67.2%)	139 (71.3%)	600 (68.1%)	

* includes 17 individuals classified as other.

The *p*-value corresponds to the comparison between “Jewish, other” and “Arab/Druze.”

^a^ Pearson’s Chi-squared test with Yates’ continuity correction.

^b^ Fisher’s Exact Test.

^c^ 33 participants (3.7%) were interviewed before or in between the time categories, these were assigned to the category closest to the date of interview.

### HRQoL After SARS-CoV-2

**FIGURE 2:**
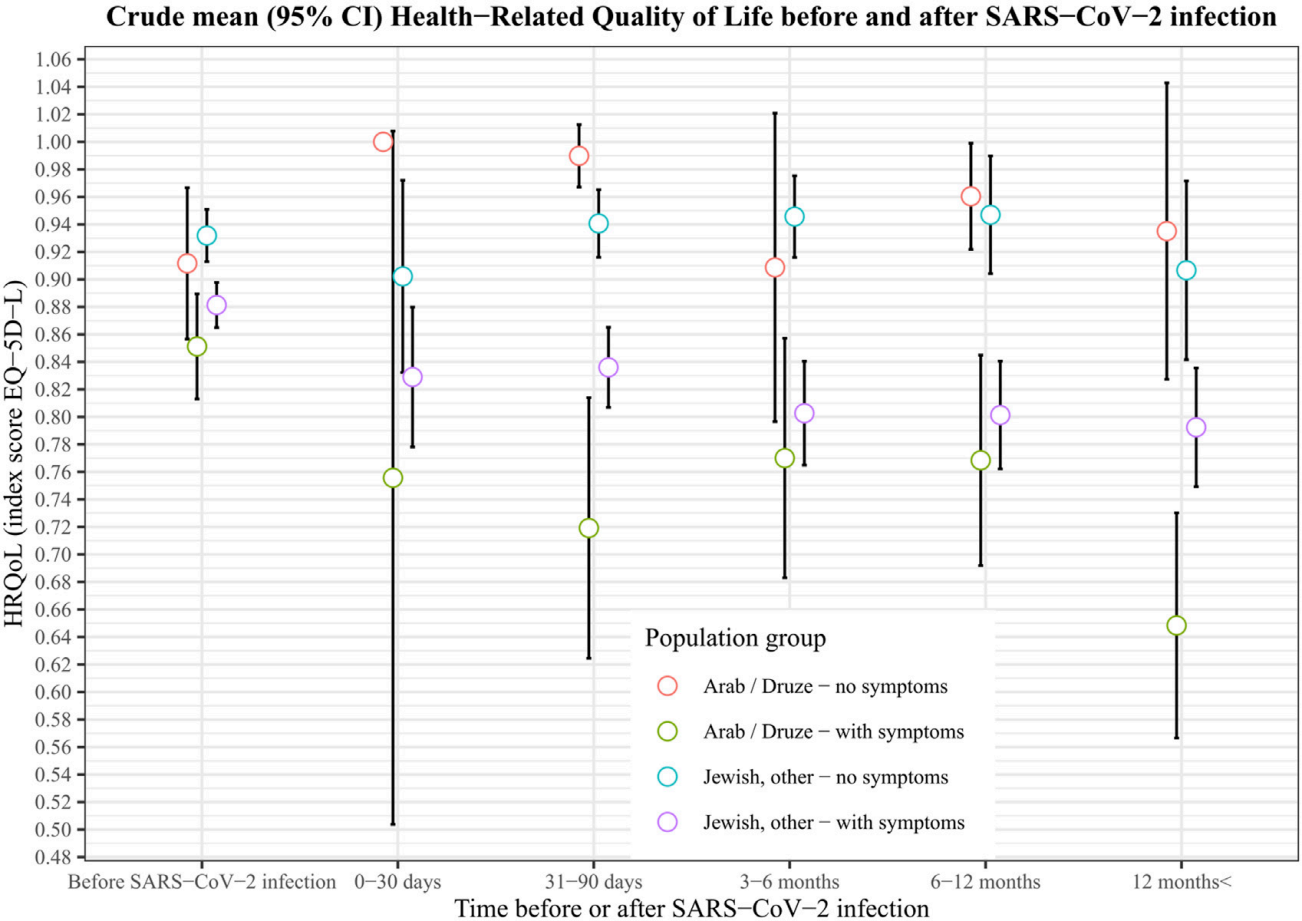
Crude mean (95% CI) health−related quality of life among Jews and Arabs/Druze before and after SARS−CoV−2 infection, Israel, 2020–22.

Overall, compared to the Jewish population the crude HRQoL-score of Arabs/Druze post-SARS-CoV-2 infection was lower at the time of the interview (0.79 vs. 0.85; *p* > 0.001). After adjusting for age, income, time of interview and place of residence, Arabs/Druze still had a lower HRQoL at the time of interview (0.83 vs. 0.88; *p* = 0.005). Figure 2 shows the crude HRQoL-scores of Arab/Druze and Jewish populations pre and post-SARS-CoV-2 infection, stratified by time elapsed since SARS-CoV-2 infection and grouped by having symptoms or not. At all time points, those reporting symptoms post-acute SARS-CoV-2 infection reported lower HRQoL scores than those who did not. HRQoL scores between Jewish and Arab populations were not significantly different within each time point up to 12 months post-infection, however, among those reporting more than 12 months post-infection, mean HRQoL was lower in the Arab/Druze population than in the Jewish one (0.72 vs. 0.83, *p* = 0.005). This was driven by a lower HRQoL among the Arab population participants who reported symptoms (Figure 2).

### Changes in Quality of Life After SARS-CoV-2

Overall, after adjusting for age, place of residence, monthly income household, date of interview and time to follow-up after SARS-CoV-2 infection, changes in HRQoL from baselines were comparable between Jews and Arabs/Druze (overall difference in HRQoL = 0.02; 95% CI: −0.02–0.06; *p* = 0.305).

**FIGURE 3:**
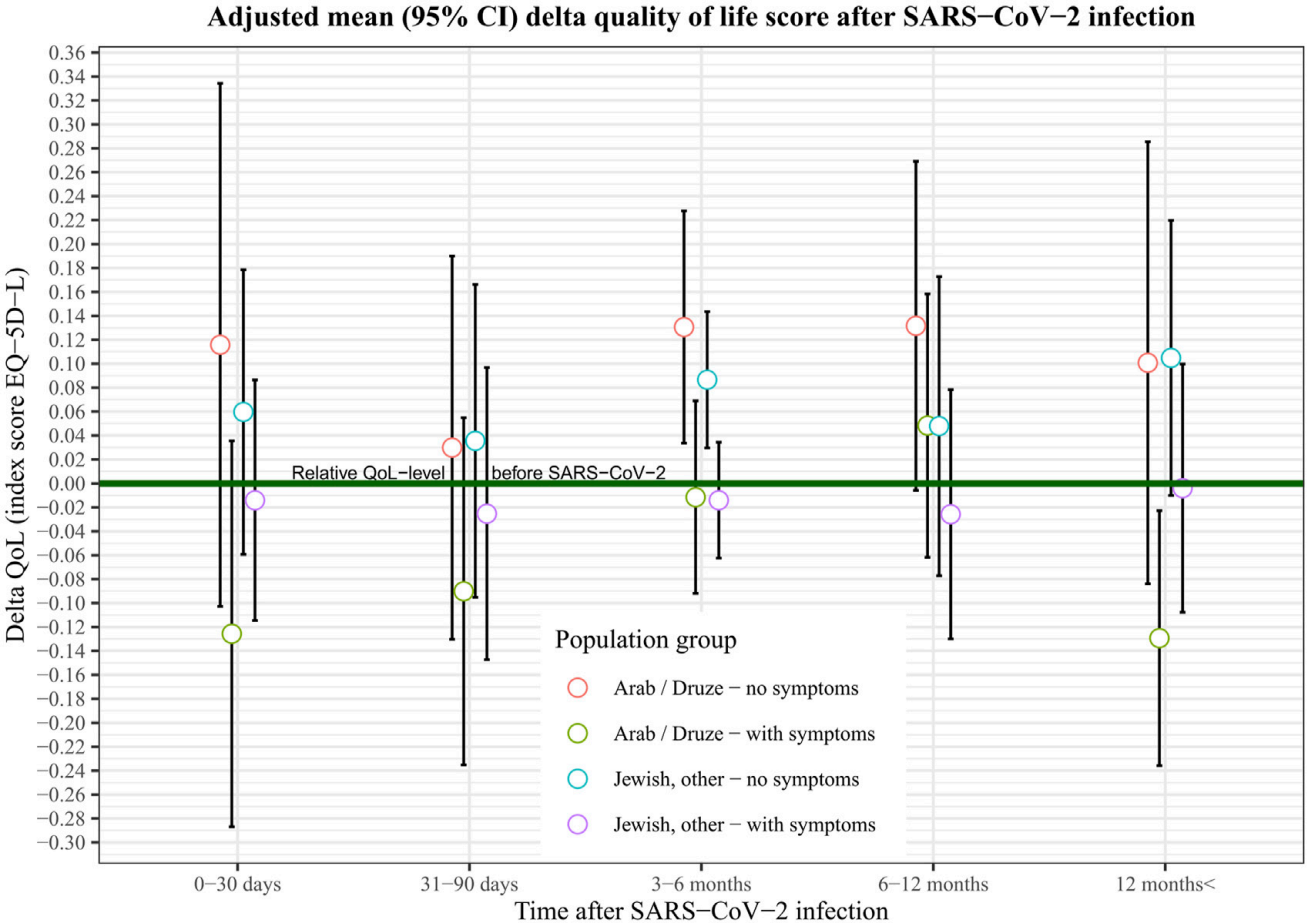
Adjusted mean (95% CI) delta quality of life score after SARS−CoV−2 infection among Jews ans Arabs/Druze, Israel, 2020–22.

When stratified by time elapsed between infection and responding to the survey there was no difference in HRQoL change from baseline between the Jewish and Arab populations among those responding within 6 months after infection (Table 2). Among participants who reported 7–12 months after infection the Arabs/Druze reported an increase of 0.08 points in their HRQoL index compared with pre-infection, while the HRQoL of Jews did not change (difference in delta 0.09, *p* < 0.05). Among those reporting more than 12 months post-infection, the Arab/Druze population reported a drop of 0.10 in their HRQoL score compared to baseline whereas the Jewish population reported very little change, the difference in change between the two groups being statistically significant (delta 0.11, *p* = 0.01, Figure 3 and Table 2).

**TABLE 2:** Comparison of delta quality of life score between Jewish and Arab/Druze population groups, stratified by time of HRQoL-measure (Israel, 2020–22).

Time of HRQoL-measure	Population group	*n*	Crude			Adjusted^a^		
			Mean HRQoL	Beta^b^ (95% CI)	*p*	Mean HRQoL	Beta^b^ (95% CI)	*p*
Before SARS-CoV-2	Arab/Druze	195	0.87			0.83		
	Jewish, other	686	0.90	0.03 (0.00, 0.06)	0.048	0.86	0.03 (0.00, 0.06)	0.059
			Mean delta HRQoL^c^			Mean delta HRQoL^c^		
0–30 days	Arab/Druze	9	−0.11			−0.07		
	Jewish, other	77	−0.04*	0.07 (−0.04, 0.19)	0.203	0.00	0.07 (−0.05, 0.19)	0.270
31–90 days	Arab/Druze	39	−0.10**			−0.06		
	Jewish, other	192	−0.05**	0.05 (0.00, 0.10)	0.0497	−0.02	0.04 (−0.03, 0.11)	0.242
3–6 months	Arab/Druze	33	−0.015			0.05		
	Jewish, other	140	−0.063**	−0.05 (−0.12, 0.02)	0.174	0.02	−0.03 (−0.10, 0.04)	0.386
7–12 months	Arab/Druze	58	0.036			0.08		
	Jewish, other	142	0.004	−0.03 (−0.11, 0.05)	0.416	−0.01	**−**0.09 (−0.17, 0.00)	0.044
12 months<	Arab/Druze	56	−0.23**			−0.10		
	Jewish, other	135	−0.09**	0.14 (0.06–0.21)	<0.001	0.01	0.11 (0.02, 0.20)	0.014

^a^ The mean HRQoL scores were adjusted for covariates age, income, time of interview, place of residence. Additionally, the follow-up strata showing the delta HRQOL were adjusted for days to follow-up (continuous variable).

^b^ Beta: the beta coefficient, here: the difference in delta HRQoL between Jewish, other compared to Arab/Druze.

^c^ Delta HRQoL: the difference of the HRQoL before and after COVID-19. A Paired *t*-test comparing mean HRQoL pre-COVID-19 and post-COVID-19 of the individual population groups per strata was performed with the crude data: * represents a *p*-value of 0.05–0.001, ** represents a *p*-value <0.001.

## Discussion

This cross-sectional study investigated the impact of SARS-CoV-2 on health inequalities in terms of HRQoL post-COVID-19 infection among the two main ethnic groups in Israel. Our unadjusted analysis showed that individuals infected with SARS-CoV-2, especially those reporting post-acute symptoms, reported decreases in HRQoL regardless of ethnicity, even among those reporting more than 12 months post-infection. After adjusting for other socio-economic factors, changes from baseline in HRQoL were similar in the Jewish and Arab populations up to 12 months post-infection. However among those reported more than 12 months since infection, the decrease in HRQoL was significantly higher among Arabs and Druze than among Jews. According to the literature, the minimally important difference (MID) of the EQ-5D, based on several clinical conditions, is a score change as small as 0.03 or more [18], and we measured a difference of 0.11 between our two populations groups, making the changes detected in our study not only statistically significant but also relevant from a public health perspective.

Our data includes both participants who reported post-acute symptoms and those who did not, with the intent of measuring HRQoL in the population as a high-level indicator that incorporates the direct effect of the viral infection and the indirect impact of the pandemic, such as restrictions, lockdowns, school closures, other factors that are likely to affect quality of life. The drop in HRQoL among the Arab population was driven by those reporting post-acute symptoms. However the disparities became apparent late after the infection, suggesting that factors other than the pathophysiology of the disease could also have contributed to the impact on HRQoL. The design of the study did not allow to determine what these factors were and could include individual and collective factors, related to pandemic measures or otherwise.

Although determining the causes of the observed differences between the two groups is outside of the scope of this study and would require a different design, community resilience possibly contributes to these differences. Indeed, evidence from the literature suggests that community resilience and collective efficacy affect HRQoL [19, 20]. Community resilience can be described as the ability of a community to mitigate and cope with changes or crises [21, 22]. Collective efficacy refers to a group’s shared belief in the capabilities of that group to produce attainments [23].

Before the emergence of the COVID-19 pandemic in Israel, studies have shown that the Arab minority in Israel had lower levels of community resilience compared to the Jewish majority [24, 25]. Research conducted during the COVID-19 outbreak in Israel suggested that Arab participants had higher levels of distress and lower levels of individual and community resilience compared to Jewish participants [26]. A community that lacks resources and has low resilience levels during routine times will probably face difficulties in maintaining its resilience over time, resulting in attrition of the community’s ability, despair, and a decreased quality of life—which would be compatible with a deterioration in the quality of life when facing a prolonged adverse situation. Decreases in community resilience and collective efficacy [27] may have contributed to increasing the vulnerability of the Arab minority in Israel, especially in the long term.

A decrease in HRQoL following a long follow-up time among patients suffering symptoms post-infection has previously been observed with other infectious diseases such as Chikungunya, a vector-borne disease that can lead to long-term post-infection symptoms comparable to those reported with COVID-19. With Chikungunya, the decrease in HRQoL was associated with a lack of coping strategies and the rise of emotional or psychological burdens when chronic symptoms did not disappear [28, 29]. The lack of coping strategies is part of lower resilience, and may have played a crucial role in the HRQoL-drop among the Arab population 12 months after the infection.

Adjusting for potential confounders reduced the difference in delta HRQoL at 12 months by only 0.03 points. This suggests that socioeconomic indicators might only explain a small extent of the difference in HRQoL between the two population groups. Although our study design does not enable to identify the root cause of HRQoL differences in the two groups included, the literature offers possible explanatory models for health disparities stemming from different circumstances between population groups that should be considered, including 1) differences in behavioural habits like exercise and diet (health-behaviour model), 2) differences in stress due to, e.g., discrimination or minority stress (psychosocial stress model), 3) genetic differences (racial-genetic model) and 4) different social genesis of perceptions regarding health-related constructs (structural-constructivist model) [30]. Literature about health disparities in Israel includes assessments during the SARS-CoV-2 pandemic and supports several of the aforementioned theories, as we describe henceforth. Arabs living in Israel were found to have higher excess mortality than Jews, which have been linked to adverse health behaviours and health conditions that are more prominent in the Arab population [31]. These adverse health behaviours include smoking, physical inactivity, and a high fat and refined cereal diet [32], leading to higher risks of, e.g., diabetes and obesity [33] and vulnerability for severe COVID-19. Another possible explanation of disparities is derived from the psychosocial model, which suggests a higher stress level leading to adverse health effects and less resilience due to institutional and perceived racism. Indeed, Arabs living in Israel perceive generally higher levels of personal and institutional discrimination than other groups, which was also during the SARS-CoV-2 pandemic related to higher levels of stress [34–36]. Although there are studies that suggest inequalities in COVID-19 susceptibility and outcomes supporting the racial-genetic model [37, 38], we found no studies that suggested an adverse or beneficial racial-genetic effect for the major Israeli population groups. Israeli initiatives in public health using a social-constructivist approach to bridge gaps between communities’ lived realities and institutions exist [39, 40], but we found no literature describing how goals and aspirations are constructed and interact with social realities and health of different population groups in Israel.

Respondents that assessed their post-COVID-19 HRQoL in summer (July–August 2021) were in all models (See Supplementary Tables S1–S6) associated with less impact on HRQoL, implying a seasonal effect where HRQoL is higher in summer, which is in accordance with the literature [17]. Even though a higher proportion of the Arab/Druze population reports in the winter, this effect was adjusted for in the analysis and therefore did not explain the observed difference.

The study suffers from a number of limitations including a recall bias of the HRQoL before SARS-CoV-2 and the self-reported nature of symptoms and quality of life. The limited response rate and the use of a digital survey that might not be accessible for certain populations may have biased our sample. Although our samples’ age and ethnicity were comparable to the general population [14], females and low-income households were over-represented. Because this analysis makes use of a single survey per participant, although participants answered after different time periods following infection, we cannot directly analyse the data longitudinally. We used hospitals from the Northern District as a sampling frame, meaning that study participants are mainly residents of Northern Israel. The distribution of the Arab population of the North differs from the overall distribution in Israel. Specifically, the proportion of Christian Arabs, and Druze, comparatively less socio-economically deprived groups, is higher than the national average, whereas the most deprived group, namely, Negev Bedouins, does not reside in this part of the country. Our study is therefore not fully representative of the national situation and may have underestimated differences between Jewish and Arab population that may exist at the national level in terms of HRQoL.

Finally, because of the observational nature of the study, we cannot directly establish causality. We could not account for individual or collective events other than the pandemic affecting quality of life.

Compared with the Jewish majority, those infected with SARS-CoV-2 among the Arab and Druze minority suffered a greater impact on HRQoL 12 months after a SARS-CoV-2 infection. Our study could not discriminate between the direct impact of SARS-CoV-2 infection and adverse effects of lockdowns or other health policies. Socioeconomic variables could not fully explain the underlying cause of this disparity between Arabs/Druze and Jews. It is therefore important to also explore other causes of health disparities that widen health inequalities between population groups in Israel. Using a health behavior-, psychosocial stress-, or a structural-constructivist model might aid in explaining health inequalities that arose during the SARS-CoV-2 pandemic between population groups in Israel. Future research should consider using the latter models to understand the cause of health disparities between population groups in Israel during the SARS-CoV-2 pandemic. The long-term impact of COVID-19 is emerging as a global and persisting public health issue. Future research should follow up the evolution of HRQoL observed in different populations groups in Israel and beyond, and its impact on inequalities beyond the follow-up time of this study.
