# Supplementary material for: Changes in Quality of Life Following SARS-CoV-2 Infection Among Jewish and Arab Populations in Israel: A Cross-Sectional Study
Source: Int J Public Health. 2023 Jun 12;68:1605970. doi: 10.3389/ijph.2023.1605970 (PMC10291044; doi:10.3389/ijph.2023.1605970)
Supplement: Supplementary file 1 [file DataSheet1.docx]

**Supplementary Tables**
Supplementary table 1. Non-stratified regression model delta HRQoL. Israel, 2020-22

| **Characteristic** | **Beta** | **95% CI** | **p-value** |
| --- | --- | --- | --- |
| **Population group** |  |  |  |
| Arab / Druze | — | — |  |
| Jewish, other | 0.02 | -0.02, 0.06 | 0.3 |
| **Place of residence** |  |  |  |
| Other | — | — |  |
| Rural | -0.04 | -0.16, 0.09 | 0.6 |
| Urban | -0.05 | -0.17, 0.08 | 0.4 |
| **Monthly income household** |  |  |  |
| <8000 NIS | — | — |  |
| >15,000 NIS | 0.03 | -0.01, 0.07 | 0.11 |
| 8000-15,000 NIS | 0.02 | -0.01, 0.06 | 0.2 |
| **Time of interview** |  |  |  |
| 2021 Jul-Aug | — | — |  |
| 2021 Nov-Dec | -0.29 | -0.34, -0.25 | <0.001 |
| 2022 Mar-May | -0.26 | -0.31, -0.22 | <0.001 |
| **Age** | 0.00 | 0.00, 0.00 | 0.069 |
| **Time to follow-up after SARS-Cov-2 infection** | 0.00 | 0.00, 0.00 | 0.006 |

Supplementary table 2. Regression model delta HRQoL 0-30 days after SARS-CoV-2 infection. Israel, 2020-22

| **Characteristic** | **Beta** | **95% CI** | **p-value** |
| --- | --- | --- | --- |
| **Population group** |  |  |  |
| Arab / Druze | — | — |  |
| Jewish, other | 0.07 | -0.05, 0.19 | 0.3 |
| **Place of residence** |  |  |  |
| Other | — | — |  |
| Rural | -0.05 | -0.38, 0.29 | 0.8 |
| Urban | -0.03 | -0.37, 0.30 | 0.8 |
| **Monthly income household** |  |  |  |
| <8000 NIS | — | — |  |
| >15,000 NIS | 0.06 | -0.03, 0.15 | 0.2 |
| 8000-15,000 NIS | 0.01 | -0.08, 0.09 | 0.9 |
| **Time of interview** |  |  |  |
| 2021 Jul-Aug | — | — |  |
| 2021 Nov-Dec | -0.24 | -0.40, -0.09 | 0.003 |
| 2022 Mar-May | -0.18 | -0.28, -0.09 | <0.001 |
| **Age** | 0.00 | 0.00, 0.00 | 0.8 |
| **Time to follow-up after SARS-Cov-2 infection** | 0.00 | 0.00, 0.01 | 0.6 |

Supplementary table 3. Regression model delta HRQoL 31-90 days after SARS-CoV-2 infection. Israel, 2020-22

| **Characteristic** | **Beta** | **95% CI** | **p-value** |
| --- | --- | --- | --- |
| **Population group** |  |  |  |
| Arab / Druze | — | — |  |
| Jewish, other | 0.04 | -0.03, 0.11 | 0.2 |
| **Place of residence** |  |  |  |
| Other | — | — |  |
| Rural | -0.07 | -0.29, 0.15 | 0.5 |
| Urban | -0.09 | -0.31, 0.13 | 0.4 |
| **Monthly income household** |  |  |  |
| <8000 NIS | — | — |  |
| >15,000 NIS | 0.05 | -0.01, 0.11 | 0.12 |
| 8000-15,000 NIS | 0.02 | -0.04, 0.08 | 0.6 |
| **Time of interview** |  |  |  |
| 2021 Jul-Aug | — | — |  |
| 2021 Nov-Dec | -0.10 | -0.41, 0.21 | 0.5 |
| 2022 Mar-May | -0.05 | -0.36, 0.26 | 0.8 |
| **Age** | 0.00 | 0.00, 0.00 | 0.090 |
| **Time to follow-up after SARS-Cov-2 infection** | 0.00 | 0.00, 0.00 | 0.8 |

Supplementary table 4. Regression model delta HRQoL 3-6 months after SARS-CoV-2 infection. Israel, 2020-22

| **Characteristic** | **Beta** | **95% CI** | **p-value** |
| --- | --- | --- | --- |
| **Population group** |  |  |  |
| Arab / Druze | — | — |  |
| Jewish, other | -0.03 | -0.10, 0.04 | 0.4 |
| **Place of residence** |  |  |  |
| Other | — | — |  |
| Rural | -0.05 | -0.10, 0.01 | 0.11 |
| Urban |  |  |  |
| **Monthly income household** | — | — |  |
| <8000 NIS | 0.02 | -0.05, 0.09 | 0.6 |
| >15,000 NIS | 0.02 | -0.04, 0.09 | 0.5 |
| 8000-15,000 NIS |  |  |  |
| **Time of interview** |  |  |  |
| 2021 Jul-Aug | — | — |  |
| 2021 Nov-Dec | -0.29 | -0.41, -0.17 | <0.001 |
| 2022 Mar-May | -0.30 | -0.43, -0.17 | <0.001 |
| **Age** | 0.00 | 0.00, 0.00 | 0.2 |
| **Time to follow-up after SARS-Cov-2 infection** | 0.00 | 0.00, 0.00 | 0.11 |

Supplementary table 5. Regression model delta HRQoL 7-12 months after SARS-CoV-2 infection. Israel, 2020-22

| **Characteristic** | **Beta** | **95% CI** | **p-value** |
| --- | --- | --- | --- |
| **Population group** |  |  |  |
| Arab / Druze | — | — |  |
| Jewish, other | -0.09 | -0.17, 0.00 | 0.044 |
| **Place of residence** |  |  |  |
| Other | — | — |  |
| Rural | -0.04 | -0.32, 0.24 | 0.8 |
| Urban | -0.03 | -0.31, 0.25 | 0.8 |
| **Monthly income household** |  |  |  |
| <8000 NIS | — | — |  |
| >15,000 NIS | 0.05 | -0.04, 0.14 | 0.3 |
| 8000-15,000 NIS | 0.00 | -0.09, 0.08 | >0.9 |
| **Time of interview** |  |  |  |
| 2021 Jul-Aug | — | — |  |
| 2021 Nov-Dec | -0.26 | -0.36, -0.16 | <0.001 |
| 2022 Mar-May | -0.23 | -0.33, -0.14 | <0.001 |
| **Age** | 0.00 | 0.00, 0.00 | 0.6 |
| **Time to follow-up after SARS-Cov-2 infection** | 0.00 | 0.00, 0.00 | 0.3 |

Supplementary table 6. Regression model delta HRQoL more than 12 months after SARS-CoV-2 infection. Israel, 2020-22

| **Characteristic** | **Beta** | **95% CI** | **p-value** |
| --- | --- | --- | --- |
| **Population group** |  |  |  |
| Arab / Druze | — | — |  |
| Jewish, other | 0.11 | 0.02, 0.20 | 0.014 |
| **Place of residence** |  |  |  |
| Other | — | — |  |
| Rural | -0.07 | -0.32, 0.18 | 0.6 |
| Urban | -0.06 | -0.31, 0.18 | 0.6 |
| **Monthly income household** |  |  |  |
| <8000 NIS | — | — |  |
| >15,000 NIS | 0.01 | -0.09, 0.11 | 0.8 |
| 8000-15,000 NIS | 0.06 | -0.03, 0.15 | 0.2 |
| **Time of interview** |  |  |  |
| 2021 Jul-Aug | — | — |  |
| 2021 Nov-Dec | -0.37 | -0.52, -0.21 | <0.001 |
| 2022 Mar-May | -0.34 | -0.50, -0.18 | <0.001 |
| **Age** | 0.00 | 0.00, 0.00 | 0.4 |
| **Time to follow-up after SARS-Cov-2 infection** | 0.00 | 0.00, 0.00 | 0.6 |
